# The Midwives Service Scheme in Nigeria

**DOI:** 10.1371/journal.pmed.1001211

**Published:** 2012-05-01

**Authors:** Seye Abimbola, Ugo Okoli, Olalekan Olubajo, Mohammed J. Abdullahi, Muhammad A. Pate

**Affiliations:** 1 National Primary Health Care Development Agency, Abuja, Nigeria; 2 Federal Ministry of Health, Abuja, Nigeria; 3 Duke Global Health Institute, Durham, North Carolina, United States of America

## Abstract

Seye Abimbola and colleagues describe and evaluate their programme in Nigeria of recruiting midwives to rural areas to provide skilled attendance at birth, which is much poorer than in urban areas.

**Summary Points**

- Maternal, newborn, and child health indices in Nigeria vary widely across geopolitical zones and between urban and rural areas, mostly due to variations in the availability of skilled attendance at birth.
- To improve these indices, the Midwives Service Scheme (MSS) in Nigeria engaged newly graduated, unemployed, and retired midwives to work temporarily in rural areas.
- The midwives are posted for 1 year to selected primary care facilities linked through a cluster model in which four such facilities with the capacity to provide basic essential obstetric care are clustered around a secondary care facility with the capacity to provide comprehensive emergency obstetric care.
- The outcome of the MSS 1 year on has been an uneven improvement in maternal, newborn, and child health indices in the six geopolitical zones of Nigeria.
- Major challenges include retention, availability and training of midwives, and varying levels of commitment from state and local governments across the country, and despite the availability of skilled birth attendants at MSS facilities, women still deliver at home in some parts of the country.

## Introduction

Nigeria, with more than 140 million people, including 31 million women of childbearing age and 28 million children under the age of five, is by far the most populous African country. However, the maternal mortality ratio (MMR) in Nigeria is 545/100,000 live births, as only one in three births in Nigeria is attended by skilled personnel, less than 20% of children are fully immunised at age one, and 36% of pregnant women do not receive antenatal care (ANC) [1]. Thus, strengthening these services is an urgent imperative.

## Midwives Service Scheme: The Rationale

The slow rate of progress in Nigeria makes the Millennium Development Goals (MDGs) targets unachievable using current strategies alone [2]. Health indices in Nigeria vary widely across geopolitical zones (See Box 1) and socioeconomic groups [3]. The northeast (NE) zone has the highest MMR: 1,549/100,000 live births compared to 165/100,000 live births in the southwest (SW). There are also urban and rural variations with MMR of 351/100,000 live births in urban areas compared to 828/100,000 in rural areas. The under-5 mortality rate of 171/1,000 live births also varies between the lowest (219/1,000 live births) and highest (87/1,000 live births) wealth quintiles. This pattern is replicated in other indices of childhood mortality. Maternal, neonatal, and child mortality rates in Nigeria are highest in the NE and northwest (NW) zones and lowest in the southeast (SE) and SW [1]. However, although the rates are lower in the SE and SW, indices in these regions still fall short of global development targets.

**Box 1. The Political Organisation of Nigeria**

Nigeria is divided into 36 states and one Federal Capital Territory (FCT), which are further sub-divided into 774 local government areas (LGAs). There are six geopolitical zones in Nigeria: north central (six states and the FCT), northeast (six states), northwest (seven states), southeast (five states), south south (six states), and southwest (six states).

These variations in health indices are influenced by the presence of tertiary hospitals, social amenities, and a population that can afford to pay for health services that in turn attract highly skilled health workers [4]. Therefore, in much of rural Nigeria, beyond issues of access, there are inadequate human resources for providing 24-hour health services in primary health care (PHC) facilities [5]. Nigeria faces a crisis in human resources for health (HRH) in the form of health worker shortages, requiring an immediate and significant increase in the number of health workers [6], or in the meantime a strategic redistribution of health workers to grossly underserved rural areas (See Box 2).

**Box 2. The Political Economy of Health Care in Nigeria**

Health services in Nigeria mirror political organisation. The federal government is responsible for tertiary care, state governments for secondary care, and the local governments (LGs) run primary care. Health financing is tied to the flow of funds from the federation account, which are shared between levels of government according to an allocation formula that keeps about half of funds at the federal level, the 36 states share a quarter, and the other quarter is distributed to the LGs. These resources are not sectorally earmarked and the states and LGs are not constitutionally required to provide budget and expenditure reports to the federal government. This results in poor coordination and integration between levels of care, giving rise to a weak and disorganised health system with widely varying patterns of outcomes. The MSS is an unprecedented emergency stop gap collaborative effort among the three tiers of government to improve maternal and child health indices in rural Nigeria.

Efforts to better reach underserved communities have been on task shifting to community health workers (CHWs) [7]. While task shifting has offered a cost-effective expansion of the overall HRH pool, skilled attendance at birth is essential to reducing the burden of maternal mortality [8]. The shortage of skilled birth attendants in rural Nigeria impacts negatively on utilisation of services by women in these areas [5]. Launched in December 2009 , the Midwives Service Scheme (MSS) was set up to address the HRH needs in rural primary care, based on the evidence that when the number of midwives increases, utilisation of services increases, women's satisfaction with care improves, and maternal and newborn mortality decrease [8],[9]. To do this, three categories of midwives were recruited as part of the MSS: the newly graduated, the unemployed, and the retired. They are posted for 1 year (renewable subject to satisfactory performance) to selected PHCs in rural communities.

## Midwives Service Scheme: The Structure

The facilities selected for the MSS were linked in an effective two-way referral system through a cluster model in which four PHC facilities with the capacity to provide basic essential obstetric care were clustered around a general hospital with the capacity to provide comprehensive emergency obstetric care. There were 815 participating health facilities: 652 PHC facilities and 163 general hospitals. Each PHC facility has four midwives to ensure 24-hour provision of skilled birth attendance at all times, as well as other maternal and child health services.

### MSS Geographical Distribution

**Figure 1:**
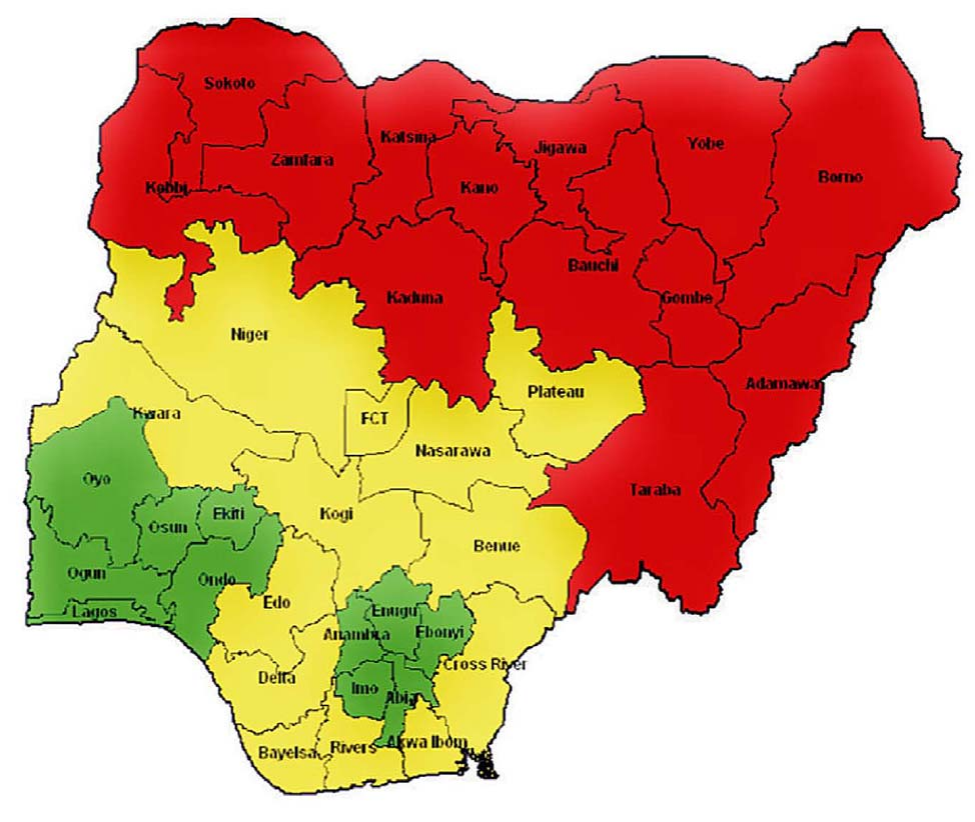
The states of Nigeria and their MMR categories. Red (northeast and northwest), very high MMR; yellow (north central and south south), high MMR; green (southeast and southwest), moderately high MMR.

The number of facilities in each of the six geopolitical zones was selected on the basis of maternal mortality burden. Nigeria was divided into three zones (Figure 1) according to MMR: very high MMR (NE and NW), high MMR (north central [NC] and south south [SS]), and moderate MMR (SE and SW). NE and NW have six clusters per state, SS and NC have four clusters per state, and SW and SE have three clusters per state. The project currently serves an estimated aggregate of 15 million people in Nigeria.

### Selection of MSS Facilities

Participating PHC facilities and general hospitals were selected based on rigorous criteria. Selected PHC facilities are in hard-to-reach areas or among underserved populations with a population of 10,000 to 30,000 people. The PHC facilities have potable water supply and offer 24-hour basic health services with minimum equipment including blood pressure apparatus, weighing scale, and basic laboratory diagnostic facilities for malaria and anaemia. Selected general hospitals provide basic services including ANC, child delivery, postnatal care, and family planning; comprehensive emergency obstetrics care and prevention of mother-to-child transmission of HIV (PMTCT) services; administration of antibiotics and intravenous fluids; and treatment of pre-eclampsia. The general hospitals have at least 12 maternity bed spaces, a functioning operating room, blood bank, and stand-by alternative power supply.

## Midwives Service Scheme: The Process

### Recruitment

The midwives under the scheme are selected with adherence to the International Confederation of Midwives (ICM) global standards for midwifery education [10]. The minimum entry level of students for midwifery education is completion of secondary education, and the minimum duration of A-Level-entry midwifery education is 3 years and 18 months for post-nursing midwifery education. The maximum age limit for recruitment is 60 years. Following an initial nationwide recruitment exercise, 2,488 (instead of the expected 2,608) successful midwives were deployed to 652 designated PHCs in the 36 states and Federal Capital Territory (FCT) on the scheme—45% of them are unemployed midwives recruited to the scheme, 44% are basic midwives during their mandatory pre-registration community service year, and 11% of them are retired midwives.

### Continuing Medical Education (CME)

To enhance the quality of their services, midwives are trained quarterly in life saving skills (LSS) and integrated management of childhood illness (IMCI). The competency-based training sessions are conducted at schools of midwifery in each state. The trainings run for 6 consecutive days and the class size varies from 24 to 32 people. The training programme involves interactive theoretical and illustrative lectures with skills demonstration and practical sessions. There are initial practical sessions on dummies, then on consenting patients in the wards towards the end of the course. Participants partake in a course review and tests to assess the effectiveness of the training.

There are no defined entry criteria for the CME, as recruitment into MSS is an ongoing process to cope with the challenge of attrition. Thus, all recruited midwives are eligible for participation in both training programmes.

### Political, Financial, and Community Commitment

Given the high level of fragmentation in the governance of the Nigerian health system (see Box 2), a crucial initiative of the MSS programme was for state and local governments to sign a memorandum of understanding with the federal government agency responsible for PHC in Nigeria, the National Primary Health Care Development Agency (NPHCDA), which is also the implementing agency for MSS. The state governments are expected to match with N20,000, the N30,000 monthly remuneration paid to the midwives by the federal government through NPHCDA.

In addition to the monthly stipend, the federal government provided basic health insurance coverage for all the midwives, provided midwifery kits for each of the participating PHC facilities and each midwife, and supplied a personal health record booklet, basic maternal and child health equipment, drugs, registers, and monitoring tools. The federal government funds the CME and provides technical support to the states and local government areas (LGAs) on the implementation, supervision, monitoring, and evaluation of MSS.

The state governments support the use of general hospitals as referral facilities for the MSS by upgrading the hospitals to provide comprehensive emergency obstetric and newborn care, including basic equipment and supplies such as drugs and other consumables, ambulance services, steady electricity and potable water supply, stationery, and security for health workers and equipment. The state governments also monitor and supervise the programme within their jurisdiction and coordinate the provision by LGAs of free decent accommodation in the host communities and at least N10,000 supplementary allowances for the midwives.

For each PHC facility, a ward development committee (WDC) made up of influential people in the community is established to enhance community participation and ownership and to promote demand for services. The WDCs meet monthly to discuss health and other developmental issues in the community under the supportive supervision of the LGAs. During the monthly WDC meetings, the midwives address any concerns of the community and brief the community on their work within the month, including their challenges. The WDCs in turn provide support to the midwives by ensuring their security and accommodation. While they do not routinely provide direct financial support for women seeking care, the WDCs support the transportation of pregnant women and neonates in cases of emergency. In addition to their clinical duties, the midwives serve as change agents in the target communities by working with WDCs to mobilise the people for health action and promoting women and child health care and home visits. Training for these roles is part of the basic midwifery training, and the midwives are involved in the creation of the WDCs.

## Midwives Service Scheme: The Outcome

### Monitoring and Evaluation Platform

MSS implementation was preceded by establishing key baseline maternal, newborn, and child health (MNCH) indicators to define goals and provide a clear framework for future evaluation. There was a nationwide survey conducted at all the facilities (primary and secondary) and communities where the intervention was located. Table 1 shows the seven core indicators of progress in the MSS, nationwide data from the Nigeria Demographic and Health Survey (NDHS) 2008, baseline data from the MSS primary care facilities, and the gains that the scheme hopes to achieve by 2015. Even though facility-based data are expected to reflect better indices, the baseline survey shows that MSS target areas are worse off compared to the national average (data from Nigerian Demographic and Health Survey 2008) even though the national data is population based.

**Table 1:** MSS core indicators and projected outcome, with data comparing 2008 NDHS with MSS facility baseline data.

MSS Core Indicators	2008 NDHS Data	MSS Baseline	5-Year Projection
PHCs with 24-h maternal health services	Not available	0%	Increase by 80%
Pregnant women with ≥4 ANC Visits	45%	39%	Increase to 80%
Deliveries by skilled birth attendants	39%	12%	Increase to 72.6%
Maternal mortality ratio (MMR)	545 / 100,000	789 / 100,000	Reduce by 60%
Neonatal mortality ratio (NMR)	46 / 1,000	11 / 1,000	Reduce by 60%
Family planning (FP) attendance	10.5%	1.02%	Increase to 50%
Children immunised in infancy	19.2%	20%	Increase by 60%

MSS, Midwives Service Scheme; NDHS, Nigeria Demographic and Health Survey.

### Impact of the MSS

**Figure 2:**
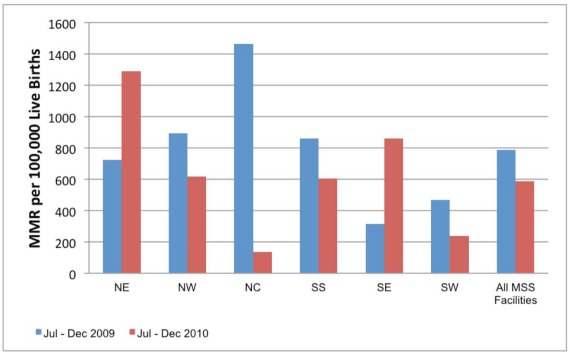
MSS facility-based maternal mortality ratios comparing July–December 2009 with July–December 2010. NE, northeast; NW, northwest; NC, north central; SS, south south; SE, southeast; SW, southwest; MSS, Midwives Service Scheme.

Figures 2–5 show MNCH indicators for the six zones comparing data from mid to the end of 2009 and mid to the end of 2010. The gains of MSS have not been even across geopolitical zones, although it shows an overall improvement in the MNCH indices.

**Figure 3 pmed-1001211-g003:**
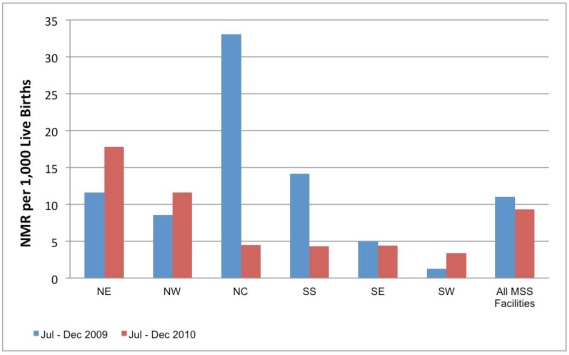
MSS facility-based neonatal mortality ratio comparing July–December 2009 with July–December 2010. NE, northeast; NW, northwest; NC, north central; SS, south south; SE, southeast; SW, southwest; MSS, Midwives Service Scheme.

**Figure 4 pmed-1001211-g004:**
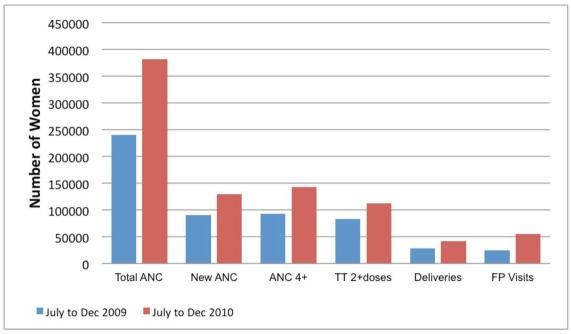
MSS facility-based maternal health indicators comparing July–December 2009 with July–December 2010. ANC, antenatal care; TT, tetanus toxoid; FP, family planning.

**Figure 5:**
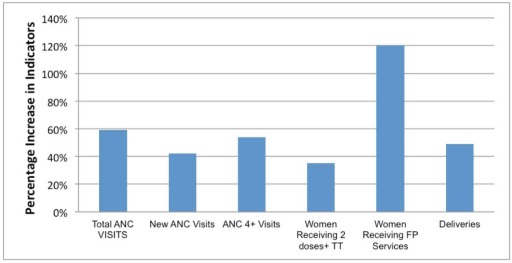
MSS facility-based maternal health indicators percentage increase from July–December 2009 to July–December 2010. ANC, antenatal care; TT, tetanus toxoid; FP, family planning.

The facility-based MMR in the same period in 2010 was 572 compared to 789 per 100,000 live births for the same period in 2009. However, facilities in the NE and SE did not show a decrease in MMR when compared to 2009. The facility-based neonatal mortality ratio (NMR) in the same period in 2010 was 9.3 per 1,000 compared to 10.97 per 1,000 live births for the same period in 2009. Facilities in the NE, NW, and SW did not show a decrease in NMR when compared to 2009. The maternal health indicators show a general overall improvement over baseline: family planning visits, pregnant women with new ANC visits and those with at least four ANC visits, facility-based deliveries, and the number of women receiving two or more doses of tetanus vaccine.

The lack of improvement in MMR and/or NMR in specific zones may be due to an increase in the proportion of high risk deliveries in the MSS PHC facilities. As shown in Figure 4, the majority of the women who attend facilities ANC still deliver at home. The additional deliveries in MSS facilities are likely to be among women with high risk pregnancy who present too late for life saving interventions in pregnancy or the neonatal period. We hope that the continued presence of skilled birth attendants in the communities will ensure positive behaviour change, especially in seeking early and routine interventions from the PHC facilities.

These data provide useful information on the progress of MSS 1 year from establishment. There have been overall improvements in the provision of MNCH services in rural areas that usually lack skilled birth attendants such as midwives. The data also provide a powerful tool for advocacy to support the scheme particularly in the NE zone where the gains have been limited.

## Midwives Service Scheme: The Challenges

- The project is currently funded from the debt relief granted to the Nigerian government by the Paris Club. The greatest threat to MSS is the uncertainty about continued funding beyond the 3-yearcommitment from the grant. However, the National Health Bill passed in 2011 promises to further provide secure funds for the administration of PHC in Nigeria [11]. The state governments are encouraged to be fully involved in MSS programmes, as the plan is for them to gradually take over the scheme in their respective states.
- Implementation of the memorandum of understanding signed with state and local governments is a persisting problem. This mainly involves provision of accommodation for the MSS midwives and irregular or delayed salary payment by state and local governments. Regular monitoring of the PHC facilities and midwives by field agents from the NPHCDA serves to coerce the state and local governments into fulfilling their roles.
- Availability of qualified midwives poses a challenge to the success of the scheme particularly in the areas of most need: the NE and NW. Ongoing recruitment and deployment of midwives to these areas are strategies employed to overcome this problem.
- Retention of midwives in the scheme is one of the major challenges. Most of the newly graduated midwives (44% of MSS midwives) are young, single, or newly married; a particularly mobile cohort who tend to return to their home zones (usually southern zones) after the completion of their 1-year mandatory pre-registration participation in the MSS. However, another set of newly graduated midwives replace the ones who leave at the end of the 1-year mandatory pre-registration programme.
- Inadequate social amenities, language barriers between the midwives and the local community, and working in hard-to-reach rural areas are some of the factors responsible for attrition. Strategies and incentives used to overcome this include attractive pay package and provision of ambulances, accommodations, and health insurance coverage for the midwives. Some hard-to-reach areas in the northern zones (NC, NE, and NW) were further provided with an additional 1,000 CHWs. Two CHWs were deployed to each facility and they provide support and complement the work of the midwives. They are also encouraged to spend time within the community to identify women and children who need care and refer appropriately. There is a long-term plan to identify and train locals to become midwives who will then work within their own communities. There are also ongoing discussions around providing supervised home delivery as part of the MSS in order to better reach women, especially in northern Nigeria, who present for ANC, but choose to deliver at home for sociocultural reasons.
- Current training of the midwives focuses mainly on LSS and IMCI. However, there is a need to also train them on other various critical aspects of health care such as PMTCT, family planning, and information and communications technology (ICT) skills. There is also a need for capacity building of the PHC team beyond just midwives.

## Conclusion

The MSS strategy of the Nigerian government recognises that strategically redistributing and improving the skill set of existing cadres of health workers is achievable on a large scale. The initiative potentially serves as a model for other developing countries within and outside sub-Saharan Africa who may need to redistribute their health workforce to reduce the inequities that exist among geographical zones and between urban and rural areas.

## Abbreviations

ANC: antenatal care
LGA: local government area
MMR: maternal mortality ratio
MNCH: maternal, newborn and child health
MSS: Midwives Service Scheme
NC: north central
NE: northeast
NMR: neonatal mortality ratio
NW: northwest
PHC: primary health care
SE: southeast
SS: south south
SW: southwest
WDC: Ward Development Committee
